# Efficacy of CDK 4/6 Inhibitors and Radiotherapy in Breast Cancer Patients with Brain Metastases

**DOI:** 10.3390/jcm12052044

**Published:** 2023-03-04

**Authors:** Marcin Kubeczko, Michał Jarząb, Aleksandra Krzywon, Donata Gräupner, Anna Polakiewicz-Gilowska, Dorota Gabryś

**Affiliations:** 1 Breast Cancer Center, Maria Sklodowska-Curie National Research Institute of Oncology, Gliwice Branch, 44-102 Gliwice, Poland; 2 Department of Biostatistics and Bioinformatics, Maria Sklodowska-Curie National Research Institute of Oncology, Gliwice Branch, 44-102 Gliwice, Poland; 3 III Department of Radiotherapy and Chemotherapy, Maria Sklodowska-Curie National Research Institute of Oncology, Gliwice Branch, 44-102 Gliwice, Poland; 4 Department of Radiotherapy, Maria Sklodowska-Curie National Research Institute of Oncology, Gliwice Branch, 44-102 Gliwice, Poland

**Keywords:** abemaciclib, palbociclib, ribociclib, CDK 4/6 inhibitors, radiotherapy, advanced breast cancer, brain metastases

## Abstract

Cyclin-dependent kinase 4/6 inhibitors (CDK4/6i) combined with endocrine therapy are the standard of care for HR-positive/HER2-negative advanced breast cancer patients. However, their role in the treatment of brain metastases is currently unclear. We retrospectively evaluate the results of patients (pts) with advanced breast cancer treated at our institution with CDK4/6i and radiotherapy to the brain. The primary endpoint was progression-free survival (PFS). Secondary endpoints were local control (LC) and severe toxicity. Among 371 pts treated with CDK4/6i, 24 pts (6.5%) received radiotherapy to the brain before (11 pts), during (6 pts), or after (7 pts) CDK4/6i treatment. Sixteen pts received ribociclib, six received palbociclib, and two received abemaciclib. Six- and twelve-month PFS was 76.5% (95% CI: 60.3–96.9) and 49.7% (95% CI: 31.7–77.9), respectively, whereas six- and twelve-month LC was 80.2% (95% CI: 58.7–100) and 68.8% (95% CI: 44.5–100), respectively. With a median follow-up of 9.5 months, no unexpected toxicity was observed. We conclude that treatment with both CDK4/6i and brain radiotherapy is feasible and should not increase the toxicity compared to brain radiotherapy or CDK4/6i alone. However, the small number of individuals treated concurrently limits the conclusions about the combination of both modalities, and the results from ongoing prospective clinical trials are eagerly awaited to understand both the toxicity profile and the clinical response fully.

## 1. Introduction

Breast cancer is the second leading cause of metastases to the central nervous system (CNS), including brain metastases (BM) and leptomeningeal metastases (LM) [1]. Although the risk of BM is highest in HER2-positive and TNBC breast cancer, they are also present in patients with HR-positive/HER2-negative (HR+/HER2−) [2]. Radiation therapy plays an essential role in the treatment of brain metastases. It might be used as whole-brain radiotherapy (WBRT) in patients with multiple metastases, or it could cover a delineated brain volume in patients with a limited number of metastases, as stereotactic radiotherapy (SRT) or radiosurgery (SRS) [3]. It is also often applied after brain metastatic lesion resection to reduce the risk of local recurrence [4].

Cyclin-dependent kinase 4/6 inhibitors (CDK4/6i) in combination with endocrine therapy are the standard of care approach in the first- or second-line setting for HR+/HER2− advanced breast cancer [5]. However, their role in the treatment of brain metastases is currently unclear since these patients were excluded from numerous pivotal clinical trials, such as MONARCH-2, MONARCH-3, MONALEESA-2, and MONALEESA-7 [6,7,8,9]. Furthermore, even in clinical trials enrolling patients with CNS involvement, this subgroup was highly under-represented, e.g., eight patients in MONALEESA-3 [10] and five patients in PALOMA-3 [11].

In contrast to the targeted therapies in HER2-positive breast cancer, there is a relative paucity of data concerning CDK4/6i activity in the brain. Abemaciclib penetrates the blood–brain barrier and achieves similar concentrations in plasma and cerebrospinal fluid. Intracranial responses were observed; however, abemaciclib did not achieve the expected intracranial objective response rates in phase II clinical trials [12]. Furthermore, palbociclib also showed intracranial activity in highly selected patients with brain metastases harboring cyclin-dependent kinase pathway alterations [13].

Multimodal treatment with local therapy remains the mainstay of the treatment; however, data concerning the safety and efficacy of such an approach in breast cancer patients treated with CDK4/6i and radiotherapy are lacking. Pre-clinical data suggested a radiosensitizing effect and a potential synergy between CDK4/6i and radiation [14], which might be associated with increased toxicity [15]. Figura et al. [16] retrospectively analyzed 15 patients treated with stereotactic radiotherapy for brain metastases with low toxicity and excellent local control.

Herein, we present safety and efficacy of CDK4/6i combined with radiation therapy in HR + HER2− breast cancer patients with CNS involvement treated at our institution.

## 2. Materials and Methods

### 2.1. Study Group

Records of consecutive patients (pts) with advanced breast cancer who received palbociclib, ribociclib, or abemaciclib in our institution were reviewed from 2018 until December 2022. Subsequently, patients who received CDK4/6i treatment and radiotherapy for the brain were enrolled in this study. The majority of patients underwent systemic treatment in the Breast Unit, while radiation therapy procedures were carried out in the Radiotherapy Department except for one patient treated with Gammaknife^®^. Thus, all data collected were derived from real-life settings, and no supplementary visits associated with the study were performed. Only patients with brain radiotherapy combined with CDK4/6i treatment sequentially within six months before CDK4/6i treatment or within 3 months after CDK4/6i completion were included. The longer time was allowed only if the patient progressed in the brain during CDK4/6i treatment but underwent neurosurgery before radiotherapy.

### 2.2. Treatment

Ribociclib, palbociclib, and abemaciclib were administered according to product specifications. The recommended dose of ribociclib is 600 mg once daily for 21 consecutive days, followed by seven days off treatment to comprise a complete cycle of 28 days, and 5 half-lives of ribociclib are 6.7 days [17]. The recommended dose of palbociclib is 125 mg once daily for 21 consecutive days, followed by seven days off treatment, resulting in a complete cycle of 28 days, and 5 half-lives of palbociclib are 6 days [18]. The recommended dose for abemaciclib is 150 mg twice daily continuously, and 5 half-lives of abemaciclib are 5.2 days [19].

CDK4/6i were combined with endocrine therapy: either letrozole or fulvestrant. Premenopausal patients received additional Luteinizing Hormone-Releasing Hormone-agonists (LHRH-agonists) for ovarian function suppression.

In the majority of patients, radiotherapy was delivered with palliative intent. Conventional (2D) radiotherapy, 3-dimensional conformal radiotherapy (3D-CRT), volumetric modulated arc therapy (VMAT), and intensity modulated radiation therapy (IMRT) with moderate hypofractionation were used. Stereotactic or radiosurgery treatment was applied in some patients. RT was performed with Varian Truebeam^®^ Linear Accelerator, Cyberknife^®^, or Gammaknife^®^.

Gross tumor volume (GTV) was defined individually, generally including the macroscopic tumor volume visible on computed tomography (CT) images fused with magnetic resonance (MRI). In the postoperative setting, no GTV was contoured, and CTV was delineated as a tumor bed. Planning target volume (PTV) was outlined, adding an adequate margin to the GTV or CTV. In stereotactic radiotherapy, CTV was equal to PTV. In patients with multiple metastases, the whole brain was contoured as a CTV; in bone and/or meningeal metastases, CTV was defined as all bones of the skull and involved dura/pia mater. In 2D radiotherapy, two opposing treatment fields were set according to bony structures. The prescribed dose was defined according to guidelines for palliative radiotherapy and personalized with respect to patients’ characteristics, such as tumor site and burden, previous treatments, and performance status. Throughout RT, each patient was monitored by a radiation oncologist at least once a week for evaluation and management of early toxicity.

The primary endpoint was progression-free survival (PFS). Secondary endpoints were local control (LC) and severe toxicity.

Response to treatment was assessed as Progressive Disease (PD), Stable Disease (SD), Partial Response (PR), or Complete Response (CR) according to Response Evaluation Criteria in Solid Tumors version 1.1 (RECIST 1.1). Progression-free survival (PFS) was defined as the time from the beginning of CDK4/6i treatment to Progressive Disease (PD) or the last date of patient follow-up. Local control (LC) in the brain was defined as the time from radiotherapy to disease progression in the brain based on MRI or CT or the last date of patient follow-up. Toxicity grade (G) was assessed with Common Terminology Criteria for Adverse Events (CTCAE) version 5.0.

### 2.3. Statistical Analysis

Categorical variables were shown as frequencies and percentages. Continuous data were summarized as median values with interquartile ranges (25% to 75%, IQR). PFS and LC were estimated using the Kaplan–Meier method, and 95% confidence intervals (CIs) for the survival curves were calculated. Survival [20], survminer [21], and survMisc [22] packages were used. All computational analysis was performed in the R environment for statistical computing version 4.0.1 “See Things Now” released on 6 June 2020 (R Foundation for Statistical Computing, Vienna, Austria, http://www.r-project.org, accessed on 20 December 2022).

## 3. Results

### 3.1. Patients and Treatment Characteristics

Among 371 advanced breast cancer patients treated with CDK4/6i at our institution, 24 pts (6.5%) received RT to the brain volume before starting CDK4/6i treatment, during this treatment, or just after CDK4/6i completion. Patients’ characteristics are shown in Table 1. Fourteen pts (58%) were irradiated for the brain metastases; within this group, four pts had both brain and skull metastases. Ten pts (42%) were irradiated for the brain volume due to skull metastases with dura/pia mater involvement of various extents, from local to extensive infiltration. In the majority of pts, brain metastases were diagnosed with MRI performed due to symptoms. In 3 pts, MRI was performed as an extension of the diagnostic after CT of the brain. In one patient, brain metastasis was found accidentally in PET CT performed to assess the stage of the disease. Almost all pts were monitored with MRI. The majority of pts had recurrent disease (18 pts, 75%). One patient had a recurrence of the disease as an isolated brain metastasis. Almost all pts had multiple bone metastases besides the skull (23 pts; 96%).

**Figure 1:**
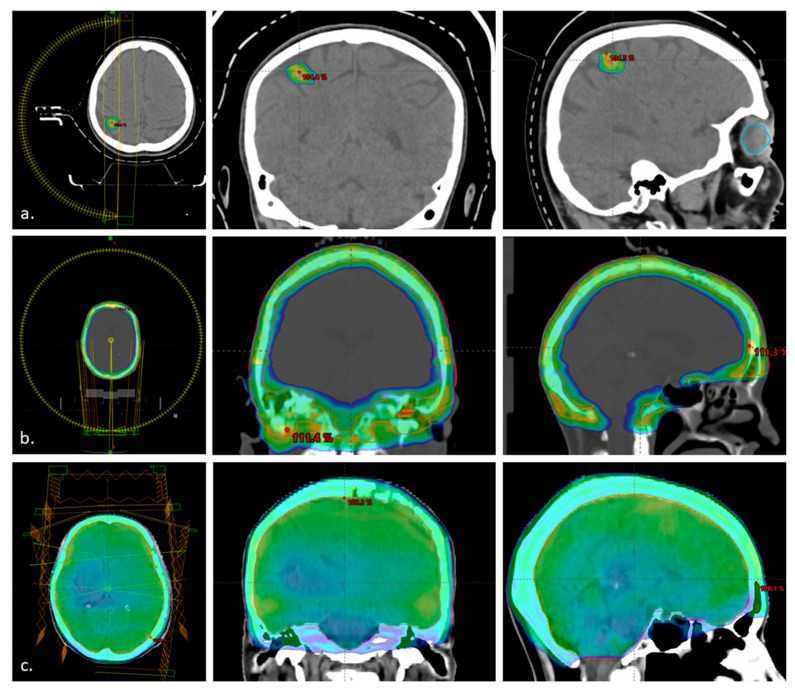
Examples of dose distribution. (**a**) Stereotactic radiotherapy to metastasis 24 Gy in 3 fractions 6X FFF (6 megavoltage flattening filter-free photon beams) photons VMAT (volumetric modulated arc therapy); (**b**) skull bone radiotherapy 20 Gy in 5 fractions 6X photons (6 megavoltage photon beams) VMAT; (**c**) whole brain radiotherapy 20 Gy in 5 fractions using 6X photons IMRT (intensity modulated radiation therapy). Left panel transvers plane, middle coronal plane, right sagittal plane. Minimum dose is set for 95%.

The median time between radiation therapy and CDK4/6i treatment was 30 days (IQR: 1–90 days). The median time from RT to CDK4/6i in the group of patients who received RT before CDK4/6i (n = 11) was 66 days (range 15–166 days). Among five patients with the most prolonged interval between RT and CDK4/6i, three patients received chemotherapy before CDK4/6i and switched to CDK4/6i without disease progression. One patient refused systemic treatment initially after RT and agreed finally after progression in the liver was identified in the following CT. The last patient’s CDK4/6i initiation was postponed due to fatigue after RT. The median time from RT to CDK4/6i in the group of pts who received RT after completing CDK4/6i (n = 7) was 14 days (range 1–139 days). A patient with the longest time interval underwent neurosurgery before RT. Six patients had RT during CDK4/6i treatment, including two patients who received CDK4/6i simultaneously with radiation therapy. One patient continued CDK4/6i (palbociclib) concomitantly during the whole radiotherapy. In the second patient, ribociclib was withheld from day 2 of RT due to dexamethasone implementation and potential drug-to-drug interactions. For the other four patients treated with ribociclib, CDK4/6i treatment was withheld before RT and restored after RT completion. Given the half-lives of palbociclib and ribociclib, the median time of concurrent CDK4/6i plus RT treatment was four days (range 1–5). Three pts were irradiated after neurosurgery. RT details are shown in Table 2. Examples of dose distribution and variable treatments is shown on Figure 1.

### 3.2. Treatment Efficacy

The median CDK4/6i cycles were 8 (range 3–57). Most patients achieved SD (17/24; 71%) during CDK4/6i treatment, 4 PR as the best clinical response, and three pts had PD without a positive response to the treatment. All patients achieving PR had regression at the first assessment three months after initiation of CDK4/6i. The main reason for CDK4/6i discontinuation was PD (15/18 pts, 83%). The liver was the leading site of disease progression during CDK4/6i treatment (eight pts). The majority of patients received a subsequent line of systemic treatment after PD on CDK4/6 pts received: fulvestrant plus alpelisib (one pt), fulvestrant monotherapy (two pts), fulvestrant plus metronomic cyclophosphamide (twopts), fulvestrant plus capecitabine (two pts), capecitabine (three pts), paclitaxel (one pt), pertuzumab plus trastuzumab plus pertuzumab (1 pt, conversion to HER2-positive subtype based on pathology from resected metastasis).

#### 3.2.1. Progression-Free Survival

**Figure 2:**
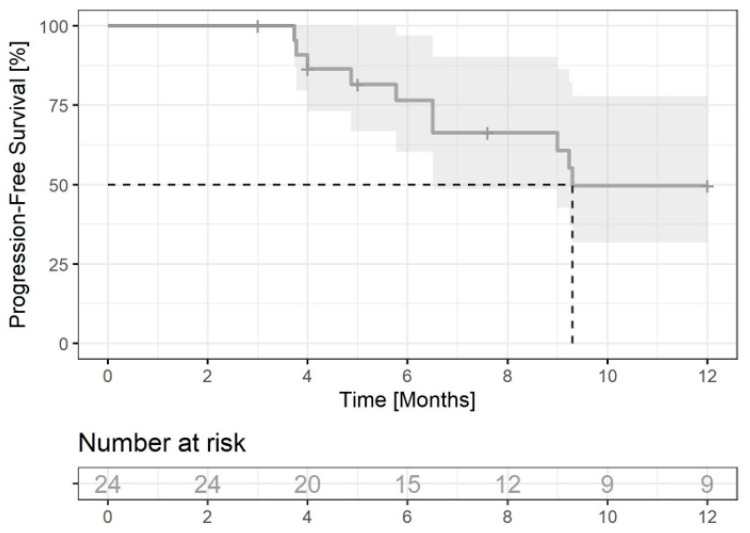
PFS of all studied patients (n = 24). The dashed line indicates median PFS, (progression-free survival) grey field indicates 95% CI.

At the time of data cut-off, 15 pts had PD, 6 continued CDK4/6i treatment, and 3 pts were lost to follow-up. Six-month PFS was 76.5% (95% CI: 60.3–96.9) and twelve-month PFS was 49.7% (95% CI: 31.7–77.9) (Figure 2). The median PFS of all studied pts was 9.3 months.

**Figure 3:**
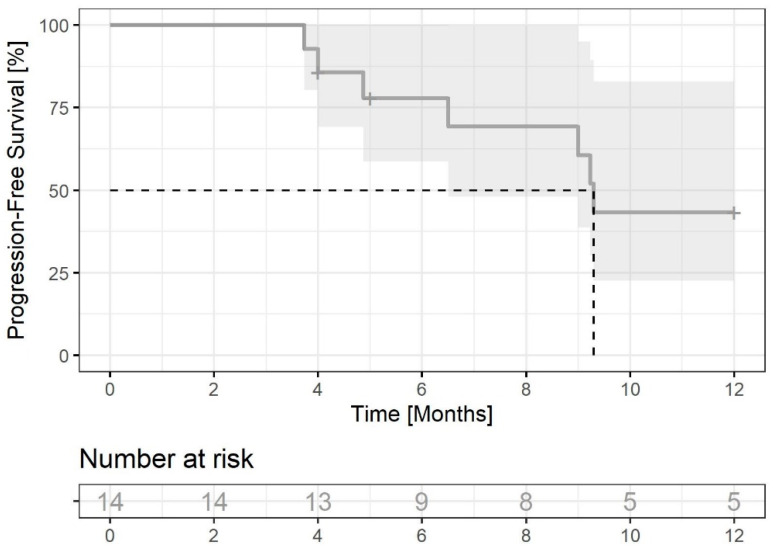
PFS of patients with brain metastases (n = 14) (excluding those with bone and meningeal metastases only). The dashed line indicates median PFS, grey field indicates 95% CI.

In the population of breast cancer patients who undergo radiotherapy for brain metastases (n = 14) (excluding those with bone and meningeal metastases only), at the time of data cut-off, ten patients had PD, three continued CDK4/6i treatment, and one was lost to follow-up. Six-month PFS was 77.9% (95% CI: 58.7–100) and twelve-month PFS was 43.3% (95% CI: 22.6–82.9) (Figure 3). The median PFS was 9.3 months. Long-term responses were observed—at the time of data cut-off, one patient continued ribociclib for 55.1 months.

#### 3.2.2. Local Control in the Brain

**Figure 4:**
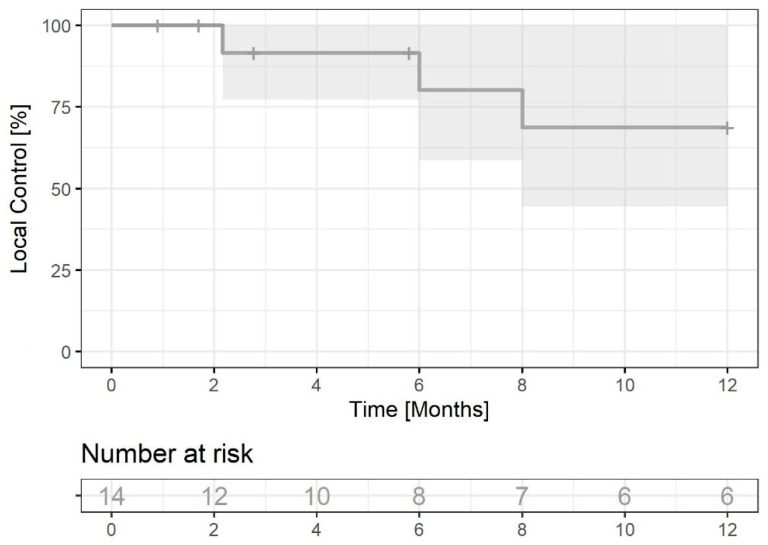
Local control in the brain (n = 14) in the group of patients with brain metastases (excluding those with bone and meningeal metastases only). Grey field indicates 95% CI.

At the time of data cut-off, three pts had PD in the brain, six pts had no PD in the brain, and five were lost from follow-up. Six-month LC was 80.2% (95% CI: 58.7–100) and twelve-month LC was 68.8% (95% CI: 44.5–100). The median LC was not reached (Figure 4). In the eight patients treated with SRS or SRT, CR was achieved in two patients treated with 24 Gy in three fractions or 25 Gy in five fractions; in another two, PR was achieved treated with 20 Gy single fraction, or 24 Gy in two fractions, and in two patients PD was recorded treated with 15 Gy in three fractions to one metastasis or 24 Gy in three fractions to three metastases. Both patients with PD had second radiotherapy first with single 6 Gy to the progressed tumour, but a patient was lost from follow up and a second patient underwent a second CK radiotherapy with 22 Gy in two fractions to the persistent two metastases, which led to further SD.

#### 3.2.3. Results of the Treatment in Patients Treated with RT before CDK4/6i Initiation

Two progressions in the brain were diagnosed in the subgroup of patients who received local treatment before CDK4/6i initiation (n = 11). At the time of data cut-off, four patients continued CDK4/6i. One patient was lost to follow-up, and six discontinued treatments due to disease progression. The main reason for CDK4/6i treatment discontinuation in this group was, similar to all the studied population, disease progression in the liver (four out of six pts). Six-month CNS control in this group was 85.7% (95% CI: 63.3–100), and twelve-month CNS control was 71.4% (95% CI: 44.7–100). However, no statistically significant differences between subgroups were found, probably due to the small number of patients.

### 3.3. Safety

The most common adverse event was neutropenia, which occurred in 83% of patients. A total of 38% of patients had CDK4/6i dose reduction (9/24), mainly due to neutropenia, and one patient had a dose reduction due to diarrhea. Three pts discontinued CDK4/6i due to worsening performance status (13%). One patient had G2 seizures during RT, one G2 headache, and one G2 vertigo. Performance status worsened in three patients. One patient had PD four months after treatment with 15 Gy in three fractions with CyberKnife. This patient had a large tumour within pons volume 16.2 cc. Moreover, the patient presented with ECOG 2 performance status and hemiparesis before radiotherapy. The second patient had a stroke and a transtrochanteric fracture of the femur. The third patient had chronic obstructive pulmonary disease exacerbation and continued letrozole monotherapy. With a median follow-up of 9.5 months (range 1–55.0 months), no other radiation-related severe toxicity was found.

## 4. Discussion

Patients with ER+/HER2− advanced breast cancer have a relatively low brain metastases incidence of 5–15%, similar to findings reported in our study [2,23]. Although relatively rare, BM still confer a poor prognosis, and the median time from diagnosis of BM to death for HR + HER− breast cancer despite multidisciplinary approach was 1.3 years [24]. The prognosis of these patients was even worse in a large multicenter real-life cohort, with median overall survival of only 7.1 months [25]. In our study, the median PFS was 9.3 months. Surprisingly, in the CompLEEment trial of ribociclib and letrozole treatment, the subgroup with CNS metastases had excellent time to progression (median not reached, 95% CI 15.5-NR), consistent with the overall study population. These patients comprised 1.6% of the studied population; more than half had very good performance status (ECOG 0), had to complete RT at least four weeks before ribociclib, and had clinically stable CNS lesions with no steroids dose increase within two weeks before the study entry. In our series, most patients were in poorer performance status. However, we also observed some long-term responses—at the time of data cut-off, one patient continued ribociclib for 55.1 months. Five patients were irradiated during CDK4/6i treatment. No new safety signals were observed. A total of 38% of patients required CDK4/6i dose reduction, similarly to the MONALEESA trials meta-analysis [26]. 

Six- and twelve-month local control rates of stereotactically treated lesions in the study performed by Figura et al. [16] were 88% and 88%, while 6- and 12-month distant brain control was 61% and 39%. In our study, 6- and 12-month intracranial control rate was lower—80.2% and 68.8%, respectively. However, 42% of our patients received whole-brain radiation therapy.

Most patients with advanced breast cancer unavoidably face disease progression due to acquired resistance to treatment. Understanding how breast tumors develop these mechanisms is crucial, and overcoming CDK4/6i resistance is an urgent challenge. Activation of the phosphatidylinositol 3-kinases (PI3Ks)/Akt/mammalian target of rapamycin (mTOR) pathway plays a vital role in cancer growth and survival and targeted therapies aimed to reverse endocrine resistance [27]. After disease progression to CDK4/6i treatment, one patient received alpelisib due to PI3K mutation. In another patient, conversion to the HER2-positive subtype was found based on pathology from resected metastasis, and treatment with HER2-targeted therapy was introduced.

Since the toxicity of RT might be augmented by other treatment modalities such as neurosurgery or chemotherapy [28], we decided to assess safety in all the studied population, despite longer time intervals between RT and CDK4/6i in some patients.

Furthermore, the risk of possible excessive adverse events associated with the combination of RT and new agents, such as targeted therapy, cannot be ignored. CDK4/6i may exacerbate early radiation toxicity as well as radiation recall. David et al. reported a case of grade 5 pneumonitis, which was postulated to be a radiation recall secondary to palbociclib, which was commenced four months after RT completion [29]. We did not observe any unexpected toxicity in our study.

In our study, only six patients received RT concurrently with CDK4/6i treatment. However, Bosacki et al. suggested that suspending CDK4/6i five half-lives before and after radiotherapy seems wiser without established safety proof [30]. Our knowledge regarding the safety of CDK4/6i inhibitors in patients who have undergone brain radiotherapy is very limited; fortunately, it is constantly increasing. Our analysis of the different treatment groups (concomitant, CDK4/6i before or after radiotherapy) showed that there is no increased toxicity of such treatment, and no recall side effects were found.

In patients undergoing CDK4/6i therapy, it is probably safe to suspend CDK4/6i just before RT initiation and resume this treatment immediately after RT completion. However, since only one patient in our cohort continued CDK4/6i concomitantly with the whole RT, further studies are needed to recommend such an approach. Given the long median duration between RT and CDK4/6i initiation in the sequentially treated patients in our study, further investigations are needed to draw adequate conclusions about the safety and optimal timing of CDK4/6i commencement after RT. Nonetheless, there are no safety concerns about RT initiation shortly after CDK4/6i completion.

The small sample size and retrospective character are the main limitations of our study. Furthermore, our studied population was heterogenous and comprised various clinical scenarios, such as patients who developed brain metastases before CDK4/6i commencement and during CDK4/6i treatment. However, this reflects daily clinical practice.

We await results from prospective studies of stereotactic radiation for brain metastases in advanced ER + HER− breast cancer patients treated with CDK4/6i. However, two trials were terminated due to slow accrual (one with palbociclib, NCT02774681; the other with abemaciclib, ribociclib, or palbociclib, NCT04585724). Abemaciclib is under investigation with an aromatase inhibitor or fulvestrant (NCT04923542), with estimated study completion at the end of 2024. Furthermore, it is also studied in combination with elacestrant, a new selective estrogen receptor degrader (NCT04791384, NCT05386108).

To our knowledge, this is the largest single institution experience on CDK4/6i and radiotherapy in HR + HER2− patients with CNS involvement. Such a combined local and systemic treatment approach seems safe and effective; however, many patients were lost to follow-up mostly due to deterioration of performance status or continuation of treatment in affiliated centers, which is one of the limitations of our study.

## 5. Conclusions

Taking into account the results of our study, we can conclude that treatment with a combination of CDK4/6i and short-course brain radiotherapy is feasible and should not increase the toxicity in comparison to brain radiotherapy or CDK4/6i alone. Nonetheless, data concerning patients treated with CDK4/6i concomitantly with RT are small.

To confirm a synergistic radio-sensitizing effect and fully understand both the toxicity profile and the clinical response, we need the results from many ongoing prospective clinical trials evaluating the safety of such combined treatment. 

## Figures and Tables

**Table 1:** Characteristics of the treated population.

Variable	n	%
No. of patients	24	100
Age (median, IQR range)	52 (41–60)	
ECOG ^1^		
0	6	25
1	14	58
2	4	17
CDK4/6i		
Ribociclib	16	67
Palbociclib	6	25
Abemaciclib	2	8
Endocrine therapy		
Letrozole	15	62
Fulvestrant	9	38
De novo disease	6	25
Previous CHT	18	75
Previous CHT < 1y ^2^	6	25
RT in relation to CDK4/6i		
Before starting CDK4/6i	11	46
Concurrent with CDK4/6i	6	25
After CDK4/6i completion	7	29

^1^ ECOG—Performance status assessed by Eastern Cooperative Oncology Group; ^2^ CHT < 1y—chemotherapy within one year before CDK4/6i treatment.

**Table 2:** Radiation therapy details.

Technique	RT Total Dose (Gy)	RT Dose per Fraction (Gy)	No. of pts n = 24 (%)
GammaKnife	20	20	1 (4%)
CyberKnife	15	5	1 (4%)
Linac Stereotactic Radiation Therapy	24	8	2 (8%)
	24	12	2 (8%)
	25	5	2 (8%)
VMAT WBRT	20	4	2 (8%)
	30	3	1 (4%)
IMRT WBRT	20	4	2 (8%)
3D WBRT	20	4	3 (13%)
2D WBRT	20	4	2 (8%)
VMAT—the base of the skull (tumour bed)	30	3	1 (4%)
VMAT Skull bone + adjacent dura/pia	20	4	4 (17%)
IMRT Retrobulbar infiltration	20	4	1 (4%)

Abbreviations: RT — radiation therapy; VMAT — volumetric modulated arc therapy; IMRT — intensity modulated radiation therapy; WBRT — whole brain radiation therapy

## Data Availability

Core set with data of patients treated with radiotherapy and iCDK4/6 is available.
